# Monitoring diaphragmatic effort during diaphragm neurostimulation-assisted ventilation

**DOI:** 10.1186/s13054-025-05810-z

**Published:** 2025-12-23

**Authors:** Andrea Castellvi-Font, Idunn S. Morris, Francisco José Parrilla-Gómez, Matías Madormo, Catherine A. Bellissimo, Viral Thakkar, Nawzer Mehta, Thiago Bassi, Laurent J. Brochard, Niall D. Ferguson, Ewan C. Goligher

**Affiliations:** 1 Critical Care Department, Critical Illness Research Group (GREPAC), Hospital del Mar, Hospital del Mar Research Institute (HMRI), Barcelona, Spain; 2 Department of Medicine and Life Sciences (MELIS), Universitat Pompeu Fabra (UPF), Barcelona, Spain; 3 Department of Medicine, Division of Respirology & Critical Care, University Health Network, Toronto, Canada; 4 Interdepartmental Division of Critical Care Medicine, University of Toronto, Toronto, Canada; 5 Department of Physiology, University of Toronto, Toronto, Canada; 6 Nepean Clinical School, Sydney Medical School, Faculty of Medicine and Health, University of Sydney, Camperdown, NSW Australia; 7 Department of Intensive Care Medicine, Nepean Hospital, Kingswood, NSW Australia; 8 Instituto Tecnológico de Buenos Aires, Buenos Aires, Argentina; 9 MBMed S.A., Buenos Aires, Argentina; 10 Lungpacer Medical USA Inc., Exton, PA USA; 11 Keenan Centre for Biomedical Research, Li Ka Shing Knowledge Institute, St. Michael’s Hospital, Toronto, Canada; 12 Institute for Health Policy, Management and Evaluation, University of Toronto, Toronto, Canada; 13 Toronto General Research Institute, Toronto, Canada; 14 Department of Medicine, University of Toronto, Toronto, Canada; 15 Toronto General Hospital, 585 University Ave, 9-MaRs-9024, Toronto, M5G 2N2 Canada

**Keywords:** Acute respiratory failure, Mechanical ventilation, Diaphragm dysfunction, Diaphragm neurostimulation, Diaphragmatic effort

## Abstract

**Rationale:**

Diaphragm neurostimulation-assisted ventilation (DNAV) can improve cardiopulmonary function during passive mechanical ventilation. However, this technique requires a reliable method to monitor and titrate diaphragmatic loading to avoid both insufficient and excessive diaphragmatic stimulation.

**Objective:**

To establish whether the reduction in airway pressure-time product (ΔPTPaw) obtained during diaphragm neurostimulation in assist control volume-controlled mode accurately quantifies the magnitude of respiratory muscle effort elicited by neurostimulation.

**Methods:**

This was a secondary analysis of the STIMULUS trial. Diaphragm neurostimulation was titrated across four levels targeting progressive occlusion pressures of 0, − 5, − 10, and − 15 cm H₂O at two PEEP levels. At each level, airway, esophageal, and gastric pressures were recorded to compute transdiaphragmatic pressure-time product (PTPdi), respiratory muscles pressure-time product (PTPmus), and ΔPTPaw, defined as the difference in airway pressure-time product between non-stimulated and stimulated breaths. Linear mixed-effects models, Bland–Altman analyses, and receiver operating characteristic (ROC) curves were used to assess agreement and discriminative ability.

**Measurements and main results:**

Twelve patients contributed 494 high-quality respiratory cycles (63% of recorded cycles). Valid Pdi data were available in nine patients. Increasing neurostimulation was associated with higher PTPdi and PTPmus and a corresponding reduction in PTPaw. ΔPTPaw was correlated with both PTPdi (R² = 0.82) and PTPmus (R² = 0.92), with good agreement observed (limits: − 4 to 44 cm H₂O·s/min for PTPdi, and − 5 to 39 cm H₂O·s/min for PTPmus). ΔPTPaw demonstrated excellent discrimination for inadequate (area under receiver operating characteristic curve, AUROC ≥ 0.94) and excessive (AUROC ≥ 0.86) diaphragmatic effort.

**Conclusions:**

ΔPTPaw is a reliable, non-invasive surrogate for monitoring diaphragm loading during DNAV under assist-controlled volume-controlled mode and may guide neurostimulation titration in mechanically ventilated patients.

**Supplementary Information:**

The online version contains supplementary material available at 10.1186/s13054-025-05810-z.

## Introduction

Mechanical ventilation (MV) is an essential life-sustaining therapy for patients with acute respiratory failure. However, it is not without harm. In addition to ventilator-induced lung injury due to excessive stress and strain [1], prolonged diaphragmatic inactivity during MV contributes to ventilator-induced diaphragmatic dysfunction [2, 3], which has been associated with delayed weaning, prolonged ventilation, and worse clinical outcomes [4, 5]. On the other hand, maintaining appropriate levels of diaphragm activity during MV is challenging, particularly in patients with severely impaired respiratory mechanics [6–9]. In this context, transvenous diaphragm neurostimulation (DNS) has emerged as a novel strategy to prevent diaphragm disuse by activating the diaphragm in synchrony with ventilator-delivered breaths [10–19]. 

The STIMULUS trial [20] recently demonstrated the feasibility, safety, and tolerability of delivering diaphragm neurostimulation-assisted ventilation (DNAV)—MV synchronized with continuous, on-demand diaphragm stimulation—in critically ill patients expected to require prolonged passive ventilation. While larger clinical trials are needed to evaluate the impact of DNAV on clinical outcomes, optimizing DNS dosing to achieve safe and effective diaphragmatic loading is essential for its clinical implementation [21]. Both insufficient and excessive diaphragmatic effort have been associated with diaphragm dysfunction and poor outcomes [4, 5, 22], underscoring the need for accurate methods to monitor and titrate diaphragmatic loading during DNAV.

The concept of using reductions in airway pressure to estimate inspiratory muscle effort has been well established in classical respiratory physiology. Early work by Marini and colleagues quantified the inspiratory workload by comparing airway and esophageal pressure changes during passive and assisted breaths in mechanically ventilated patients [23, 24], while Macklem et al. demonstrated optimization of respiratory muscle relaxation using similar pressure-based measurements [25]. Building upon this classical measurement approach, we reasoned that its application could be particularly appropriate in the context of DNAV, as diaphragm neurostimulation elicits a diaphragmatic contraction that produces pleural and airway pressure swings similar to those observed during spontaneous inspiration.

We hypothesized that the reduction in airway pressure during stimulated breaths, expressed as the difference in airway pressure-time product between passive non-stimulated and stimulated breaths during assist control volume-controlled mode (ΔPTPaw), could serve as a reliable, non-invasive surrogate for estimating diaphragmatic muscular loading. We tested this hypothesis in a secondary analysis of data collected in the STIMULUS trial. Portions of these results have been previously presented in abstract form [26]. 

## Methods

### Study design and setting

This study is a secondary analysis of data collected during the STIMULUS trial (NCT05465083), a phase I single-arm clinical trial conducted at a quaternary academic intensive care unit (Toronto General Hospital, Toronto, Canada) [20]. The trial evaluated the safety and feasibility of delivering continuous, on-demand DNAV in patients with acute respiratory failure. Regulatory approval was obtained from Health Canada, and ethical approval was granted by the University Health Network Research Ethics Board (22-5815) prior to study initiation. A total of 19 patients were enrolled and received DNAV. Neurostimulation was delivered using a specialized central venous catheter with embedded electrodes (Lungpacer^®^ AeroPace™ Protect System; Lungpacer Medical Inc., Vancouver, Canada), positioned in the superior vena cava. Details on the study population and methods have previously been reported in detail [20]. 

### Experimental procedures

After initiation of DNAV, a nested, randomized cross-over physiological study was performed to assess the effects of varying levels of diaphragm neurostimulation. This assessment was conducted shortly after patients met criteria to initiate stimulation and successful electrode mapping had been confirmed.

DNS was delivered at four stimulation intensities: baseline (no stimulation; airway occlusion pressure, Pocc = 0 cm H₂O), and 3 levels of stimulation targeting Pocc values of − 5, − 10, and − 15 cm H₂O, respectively referred to as “low”, “intermediate”, and “high”. Each stimulation level was tested at two positive end-expiratory pressure (PEEP) levels (high and low), and each step was maintained for 10 min before data acquisition.

At each stimulation step, airway pressure (Paw), flow, esophageal pressure (Pes), and gastric pressure (Pga) were recorded using a signal acquisition system (FluxMed, MBMed, Buenos Aires, Argentina). Esophageal and gastric pressures were measured with a nasogastric catheter equipped with balloon sensors (NutriVent™, Mirandola, Italy). Transdiaphragmatic pressure (Pdi) was computed by subtraction of Pes from Pga (Pdi = Pga – Pes). The pressure generated by respiratory muscles (Pmus) was estimated by calculating the passive chest wall elastic recoil pressure using measured chest wall elastance and subtracting Pes from this value [27]. 

**Fig. 1:**
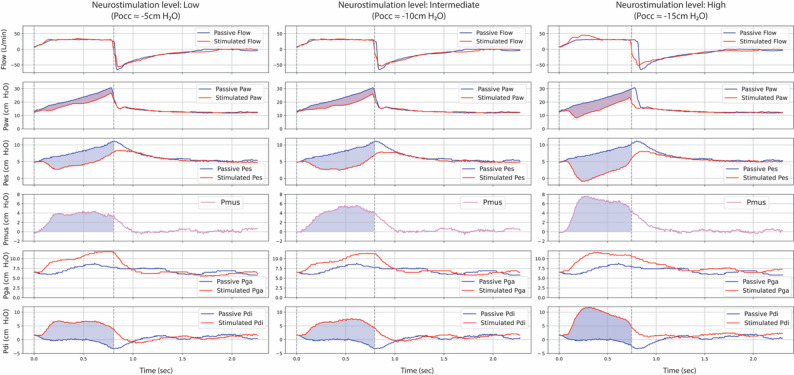
Representative respiratory waveforms across increasing neurostimulation levels during DNAV. Representative examples from a single patient showing flow, airway pressure (Paw), esophageal pressure (Pes), gastric pressure (Pga), transdiaphragmatic pressure (Pdi), and estimated muscular pressure (Pmus) tracings at three levels of diaphragm neurostimulation—low (Pocc ≈ − 5 cm H₂O), intermediate (Pocc ≈ − 10 cm H₂O), and high (Pocc ≈ − 15 cm H₂O)— each compared against a preceding passive (non-stimulated) breath. Blue shaded areas represent the pressure-time products (PTP) for each variable. The pink shared area represents the reduction in airway PTP (∆PTPaw), defined as the area between Paw curves during passive and stimulated breaths. As stimulation intensity increases, larger reductions in Paw are observed, along with corresponding increases in Pmus and Pdi, indicating progressively greater diaphragmatic effort

Pressure-time product (PTP) was calculated for Paw, Pdi, and Pmus at each stimulation step. For each variable, PTP per breath was defined as the area under the pressure-time curve during inspiration, reflecting the muscular effort generated for a single breath. PTP per minute was computed by multiplying PTP per breath by the respiratory rate (RR) at the corresponding stimulation step, thus estimating the cumulative diaphragmatic or respiratory muscle workload over time [28]. The change in airway pressure-time product (∆PTPaw) was defined as the difference between Paw-PTP measured during passive versus stimulated breaths (Fig. 1). Recordings were analyzed offline using custom software (Program for Advanced Analysis of Respiratory Mechanics [PADMER], Intellectual Property [IP]-protected; Barcelona, Spain). Additional methodological details are provided in the Online Supplement.

### Statistical analysis

Descriptive statistics are reported as means with standard deviations, medians with interquartile ranges, or counts with proportions, as appropriate. The association between ∆PTPaw and both PTPdi and PTPmus was evaluated using linear mixed-effects models. Agreement between ∆PTPaw and PTPdi or PTPmus was assessed using the Bland–Altman method, accounting for repeated measures. Linear mixed-effects models were computed to assess whether physiological factors—including lung resistance (Rlung, calculated as Δ(Paw–Pes)/inspiratory flow), lung elastance (EL, ΔPL/VT), abdominal elastance (Eab, ΔPga/VT), and insufflation time (Ti, duration of inspiratory flow)—as well as stimulation level modified the difference between ∆PTPaw and PTPdi, with patient included as a random intercept to account for within-subject correlation. To assess the discriminative performance of ∆PTPaw in detecting excessive or low diaphragmatic effort, receiver operating characteristic (ROC) curves were constructed, and the corresponding area under the curve (AUC) was calculated. Excessive effort was defined as PTPmus or PTPdi > 100 cm H₂O·s·min⁻¹, and low effort as < 40 cm H₂O·s·min⁻¹ [29]. 

All statistical analyses were performed using R (version 4.3.0; R Foundation for Statistical Computing, Vienna, Austria) and Python (version 3.12.1, Python Software Foundation, Wilmington, USA), with the following packages: *pandas*, *NumPy*, *SciPy*, and *matplotlib*.

## Results

### Study population

In the STIMULUS trial, 20 patients were enrolled and DNAV was initiated in 19. Among these, 16 underwent the nested titration study but only 12 had complete and analyzable respiratory waveform recordings. The remaining four patients were excluded due to non-interpretable waveform recordings resulting from signal acquisition issues. Baseline characteristics of the included patients are summarized in Table 1. Participants were predominantly male (75%), with a median age of 61 (IQR 48–64). Most were admitted to the ICU after pulmonary thromboendarterectomy (6, 50%) and had pre-existing lung disease at baseline (9, 75%). PaO_2_/FiO_2_ during passive ventilation (prior to stimulation) was median 283 (IQR 138–341), with normalized respiratory system elastance measuring 1.9 (IQR 1.6–2.4) cm H_2_O/(ml/kg). The majority of participants were receiving vasoactive support (82%).

**Table 1:** Baseline characteristics of the studied cohort

Characteristic	Distribution (*n* = 12)
Age (years)	61 [48, 64]
Sex: male	9 (75%)
Body mass index (kg/m^2^)	26.4 [24.3, 33.4]
Admission diagnosis	
Group 1: Acute hypoxemic respiratory failure	3 (25%)
Bacterial pneumonia	1 (8%)
Viral pneumonia	1 (8%)
Aspiration pneumonia	1 (8%)
Group 2: Post pulmonary thrombo-endarterectomy	6 (50%)
Group 3: Post-lung transplantation	3 (25%)
Preexisting respiratory disease	
Restrictive lung disease	3 (25%)
Obstructive lung disease	2 (17%)
Chronic thromboembolic disease	7 (58%)
Other	3 (25%)
Mixed pathology (two or more of above)	2 (17%)
None	1 (8%)
Physiological characteristics	
APACHE II	29 [24, 32]
SOFA	10 [10, 11]
PaO_2_/FiO_2_ (mm Hg)	284 [138, 342]
Normalized respiratory system elastance (cm H_2_O/[mL/kg predicted body weight])	1.9 [1.6, 2.4]
Lactate (mmol/L)	1.3 [1.0, 1.5]
Ventilator settings at enrolment	
Mode of ventilation	
Assist control volume control	12 (100%)
Tidal volume (mL/kg predicted body weight)	6.2 [5.9, 7.1]
Respiratory rate (min^− 1^)	20 [20, 23]
Plateau pressure (cm H_2_O)	17 [15, 21]
Positive end-expiratory pressure (cm H_2_O)	10 [7, 10]
Clinical management at enrolment	
Neuromuscular blockade infusing	1 (8%)
Inhaled nitric oxide	1 (8%)
New renal replacement therapy	0 (0%)
Extracorporeal membrane oxygenation	0 (0%)
Vasoactive agents infusing	
0	0 (0%)
1	10 (82%)
≥ 2	2 (8%)

### Respiratory tracing quality

A total of 781 respiratory cycles were collected. Two independent reviewers (ACF and FPG) assessed the quality of each cycle. Of these, 494 cycles (63.3%) were classified as high-quality and included in the final analysis. Due to challenges in acquiring reliable gastric pressure signals, valid Pdi data were available for 209 cycles (from nine patients). Detailed tracing selection and quality assessment methodology is provided in the Online Supplement.

### Diaphragmatic muscle loading during diaphragmatic neurostimulation

**Fig. 2:**
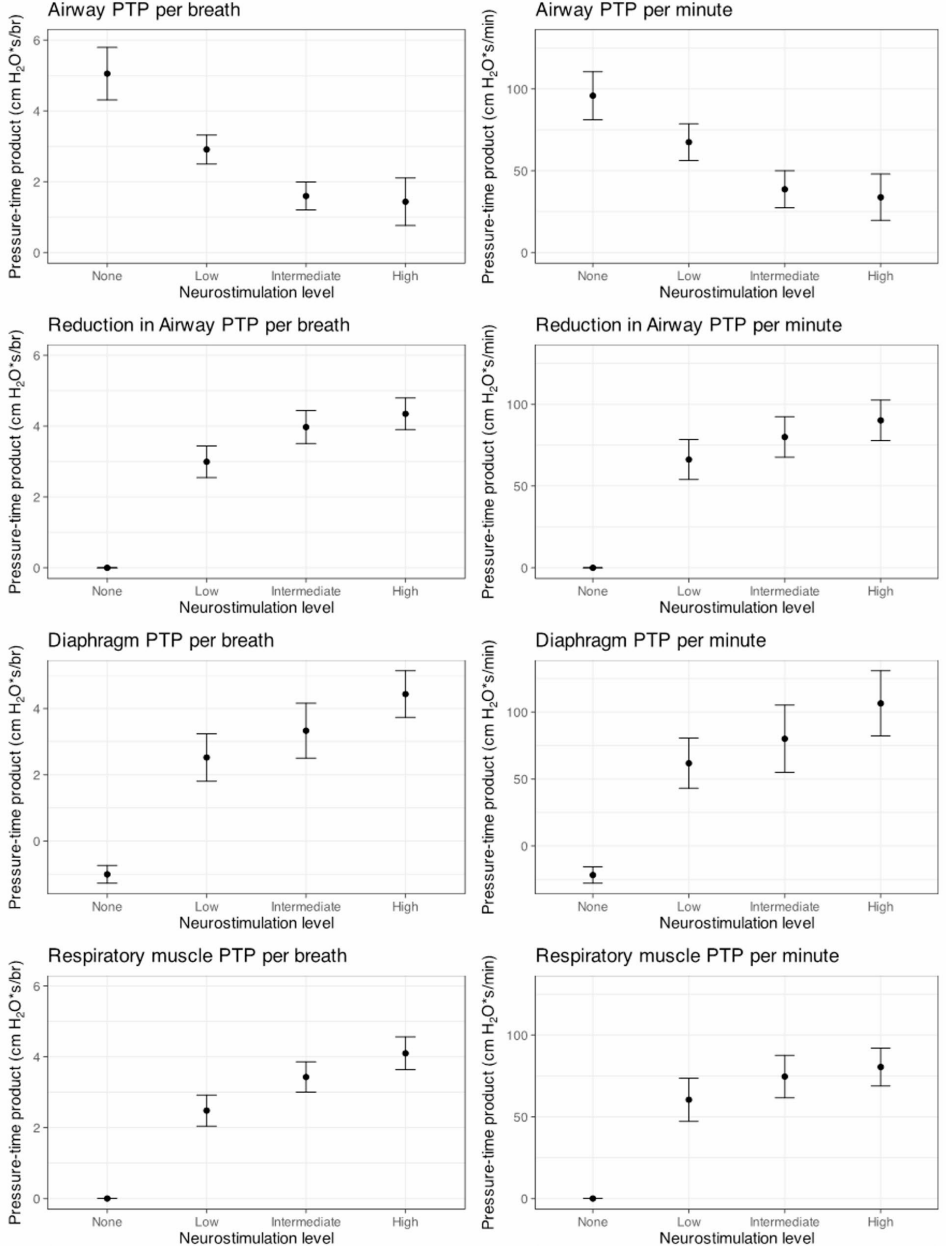
Diaphragmatic loading and airway pressure-time product across levels of diaphragm neurostimulation. Pressure-time product (PTP) values per breath (left panels) and per minute (right panels) for airway pressure (top row), reduction in airway pressure (second row), transdiaphragmatic pressure (third row), and respiratory muscle pressure (bottom row) across four neurostimulation intensities: none, low (Pocc − 5 cm H₂O), intermediate (Pocc − 10 cm H₂O), and high (Pocc − 15 cm H₂O)

Progressive increases in DNS intensity—targeting occlusion pressures (Pocc) of − 5 cm H₂O (low), − 10 cm H₂O (intermediate), and − 15 cm H₂O (high)—were associated with corresponding increases in ∆PTPaw, PTPdi, PTPmus, and decrease in PTPaw (Fig. 2). In a minority of patients, the targeted occlusion pressure of − 15 cmH₂O was not fully achieved despite maximal allowable stimulation amplitude; however, all stimulated breaths remained within the predefined upper stimulation range and were included in the analysis.

Neurostimulation resulted in a dose-dependent increase in PTPdi and PTPmus, and a corresponding reduction in airway PTP, reflected by increasing ΔPTPaw (difference in aiway pressure-time product). The non-linear relationship between neurostimulation level and PTP is consistent with observed patterns in measured Pocc across stimulation steps. This likely reflects variability in the achieved inspiratory load at higher stimulation levels, rather than a loss of measurement accuracy. Values are presented as means with standard errors.

**Fig. 3:**
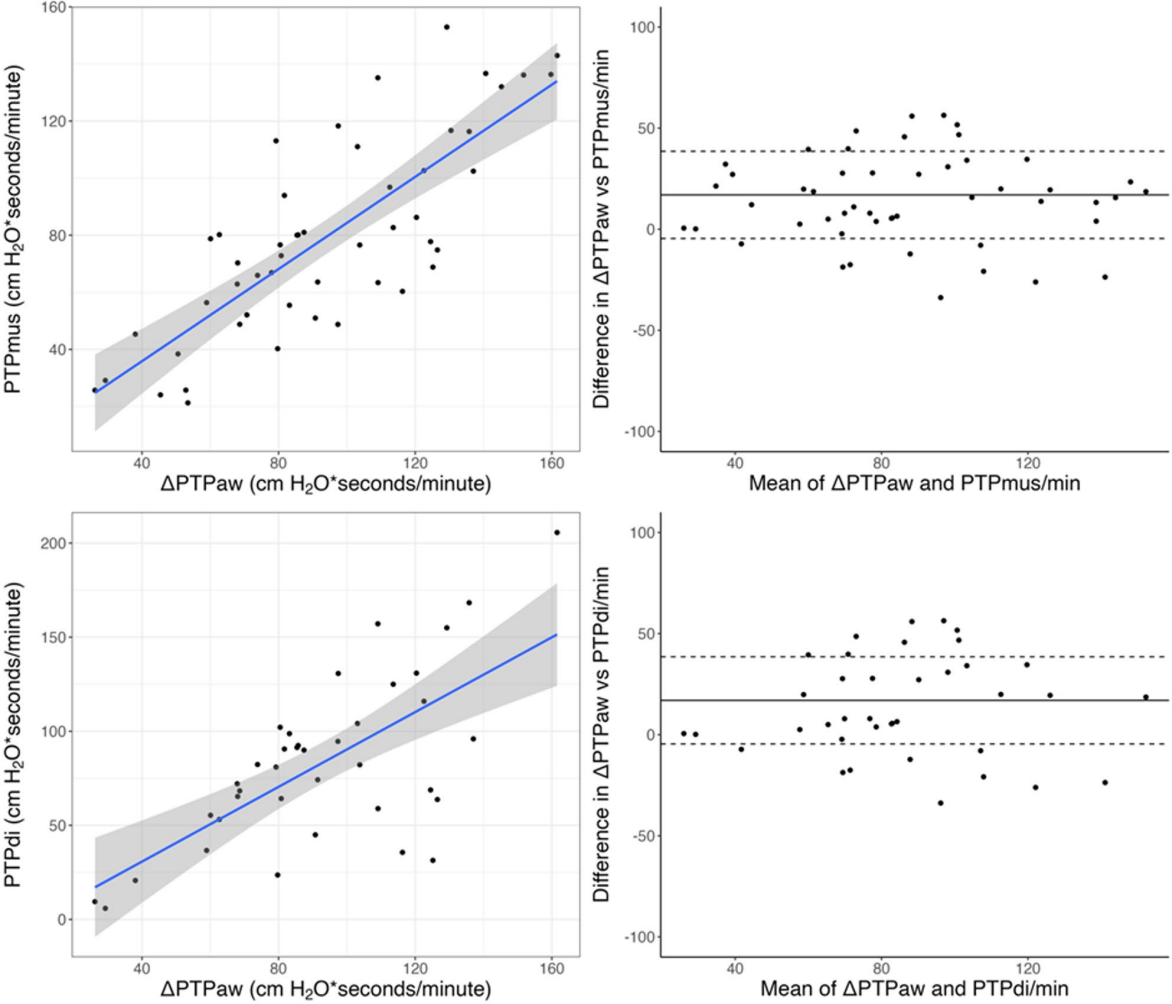
Correlation and agreement between ΔPTPaw and physiological markers of diaphragmatic effort. Relationship and agreement between the reduction in airway pressure-time product (ΔPTPaw) and respiratory muscle (PTPmus) or diaphragmatic (PTPdi) pressure-time products, all expressed in cm H₂O·s/min. Upper panels: ΔPTPaw vs. PTPmus. The left panel shows the correlation between ΔPTPaw and PTPmus per minute; each point represents a stimulation step from an individual patient. The blue line indicates the linear regression fit with 95% confidence interval (gray shading). ΔPTPaw showed a strong correlation with PTPmus (β = 0.82; 95% CI, 0.76–0.89; R² = 0.92; *p* < 0.001). The right panel displays the corresponding Bland–Altman plot, with the solid line indicating mean bias and dashed lines representing the 95% limits of agreement (bias of 20, limits − 5 to 39 cm H₂O·s/min)

∆PTPaw per breath showed a strong correlation with both PTPdi per breath (β = 1.05; 95% CI, 0.92–1.17; marginal R² = 0.79; conditional R² = 0.85; *p* < 0.001) and PTPmus per breath (β = 0.83; 95% CI, 0.67–0.98; marginal R² = 0.52; conditional R² = 0.88; *p* < 0.001). Similarly, ∆PTPaw per minute correlated with PTPdi per minute (β = 0.87; 95% CI, 0.57–1.17; marginal R² = 0.33; conditional R² = 0.82; *p* < 0.001) and PTPmus per minute (β = 0.82; 95% CI, 0.76–0.89; marginal R² = 0.86; conditional R² = 0.92; *p* < 0.001) (Fig. 3). The association between ∆PTPaw and PTPdi remained consistent across PEEP levels, with no significant interaction observed per breath (*p* = 0.765) or per minute (*p* = 0.826).

Lower panels: ΔPTPaw vs. PTPdi, structured analogously. ΔPTPaw also correlated with PTPdi (β = 0.87; 95% CI, 0.57–1.17; R² = 0.82; *p* < 0.001), with good agreement (bias of 17, limits − 4 to 44 cm H₂O·s/min).

Bland-Altman analysis demonstrated good concordance between ∆PTPaw and physiological indices of diaphragmatic effort. ∆PTPaw/breath vs. PTPdi/breath was 1.0 cm H₂O·s/breath (limits of agreement: − 0.1 to 2.0 cm H₂O·s/breath), and vs. PTPmus/breath was 0.7 cm H₂O·s/breath (limits of agreement: − 0.2 to 1.7 cm H₂O·s/breath). At the minute level, ∆PTPaw/minute showed a bias of 20 cm H₂O·s/min vs. PTPdi/minute (limits: − 4 to 44 cm H₂O·s/min) and 17 cm H₂O·s/min vs. PTPmus/minute (limits: − 5 to 39 cm H₂O·s/min) (Fig. 3).

### Reduction in airway pressure-time product to detect excessive or insufficient diaphragmatic muscle loading

∆PTPaw/min demonstrated excellent discriminative performance for detecting both low and excessive diaphragmatic effort. For low effort, defined as PTPdi/min < 40 cm H₂O·s/min and PTPdi/breath < 2 cm H₂O·s/breath [29], area under receiver operating characteristic curves (AUROCs) were 0.94 (95% CI, 0.87–1.00) and 0.84 (95% CI, 0.68–0.89), respectively. For the corresponding thresholds using PTPmus (PTPmus/min < 40 cm H₂O·s/min and PTPmus/breath < 2 cm H₂O·s/breath), AUROCs were 1.00 (95% CI, 0.99–1.00) and 0.99 (95% CI, 0.84–1.00), respectively.

**Fig. 4:**
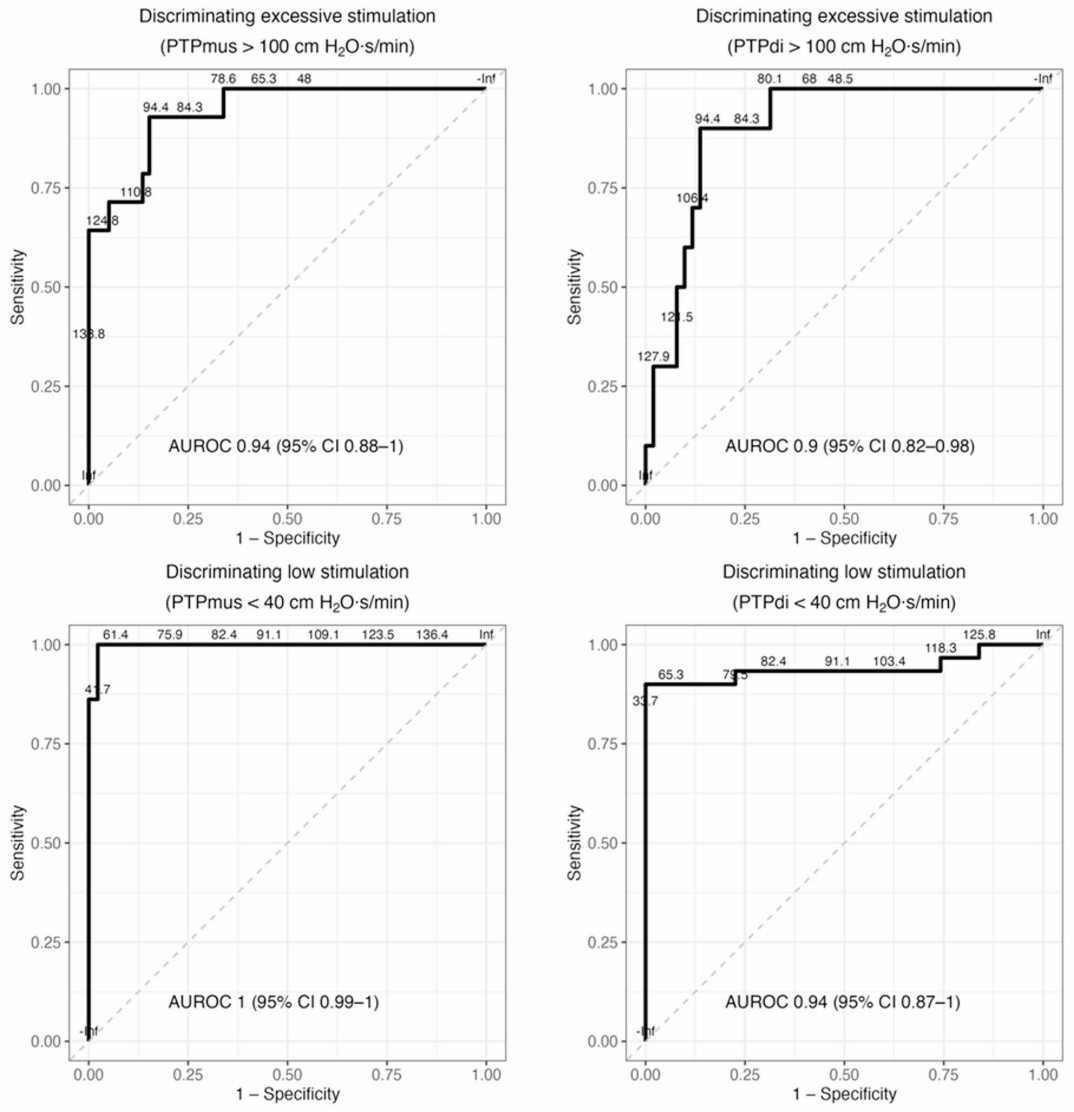
Discriminative performance of ΔPTPaw for identifying low and excessive diaphragmatic effort. Receiver operating characteristic (ROC) curves evaluating the ability of ΔPTPaw to discriminate between excessive (top panels) and low (bottom panels) respiratory effort, as assessed by respiratory muscle pressure-time product (PTPmus, left) and transdiaphragmatic pressure-time product (PTPdi, right). Excessive effort was defined as > 100 cm H₂O·s/min and low effort as < 40 cm H₂O·s/min. ΔPTPaw demonstrated excellent discriminative accuracy for both ends of the physiological spectrum, with area under the curve (AUROC) values ranging from 0.90 to 1.00

For excessive effort, defined as PTPdi/min > 100 cm H₂O·s/min and PTPdi/breath > 4 cm H₂O·s/breath, AUROCs were 0.90 (95% CI, 0.82–0.98) and 0.89 (95% CI, 0.72–0.97), respectively. Discrimination was similarly high for PTPmus/min > 100 cm H₂O·s/min (AUROC = 0.94; 95% CI, 0.88–1.00) and PTPmus/breath > 4 cm H₂O·s/breath (AUROC = 0.86; 95% CI, 0.70–0.96) (Fig. 4).

### Determinants of bias between ΔPTPaw and measures of diaphragmatic effort

A longer insufflation time (Ti) was associated with a greater bias between PTPaw/breath and PTPdi/breath (β = 6.1 cm H₂O·s/breath per second; 95% CI, 0.9 to 11.3; *p* = 0.022), while higher lung resistance (RLung) showed a non-significant trend toward increased bias (β = − 0.2 cm H₂O·s/breath per cm H₂O·s/L; 95% CI, − 0.4 to 0.0; *p* = 0.095). None of the other predictors, including stimulation level, lung elastance (EL) or abdominal elastance (Eab), were associated with bias between ∆PTPaw and PTPdi.

In contrast, for the comparison between ΔPTPaw vs. PTPmus, higher RLung was the only significant predictor of increased bias (β = − 0.3 cm H₂O·s/breath per cm H₂O·s/L; 95% CI, − 0.4 to − 0.1; *p* = 0.001). No significant effects were observed for EL, Ti, stimulation level, or Eab (e-Table 1).

## Discussion

In this physiological study nested within the STIMULUS trial, we demonstrate that ΔPTPaw, a non-invasive surrogate derived from standard ventilator signals, correlates strongly with both PTPdi and PTPmus and accurately discriminates low and excessive diaphragmatic loading during diaphragm neurostimulation. These findings suggest that ΔPTPaw may offer a practical and reliable method to monitor and titrate diaphragmatic loading in critically ill patients receiving DNAV during passive ventilation in a volume-cycled mode. Although the ‘dose’ of diaphragm neurostimulation is determined by both stimulation parameters (e.g., amplitude, frequency, and pulse duration) and the resulting diaphragmatic load, the present study focused specifically on the physiological output—diaphragmatic effort—as the clinically relevant target for monitoring.

Our findings align with classical respiratory physiology studies showing that reductions in airway pressure during inspiration can serve as a surrogate for respiratory muscle effort [25]. While this approach has been previously used to assess respiratory muscle relaxation and effort in different mechanical ventilation settings, its application in the context of DNAV is novel. The strong correlation observed between ΔPTPaw and both PTPdi and PTPmus supports the physiological validity of this classical measurement in a modern therapeutic application.

Although ΔPTPaw correlated well with both PTPdi and PTPmus, the association was notably stronger with PTPmus. This may be explained by the physiological basis of each measure. The drop in airway pressure during stimulated breaths reflects the decrease in pleural pressure generated by diaphragmatic contraction. PTPmus, which is derived from esophageal pressure (a surrogate for pleural pressure) plus the elastic recoil of the chest wall [27], more directly reflects changes in pleural pressure during inspiration. In contrast, PTPdi represents the pressure difference between the abdominal and thorax (Pga – Pes) and is influenced not only by diaphragm contraction but also by abdominal compliance and the degree of abdominal wall displacement during inspiration [30]. As a result, ΔPTPaw more closely mirrors PTPmus, which better reflects global pleural pressure dynamics, rather than PTPdi, which is more affected by trans-abdominal mechanics.

Several risks of diaphragm neurostimulation motivate the need for close monitoring. First, excess diaphragm contractions could contribute to increase lung stress and strain and injury. There is also a possible risk of load-induced diaphragm injury, particularly in the context of sarcolemmal hyperfragility and diaphragm myofibrillar hibernation [31, 32]. Excessive mechanical loading—especially when unopposed by accessory muscle recruitment—may pose a risk of diaphragmatic myotrauma [5]. Taken together, these observations underscore the need for careful titration of diaphragm stimulation to avoid excessive effort while preserving sufficient activity to prevent disuse.

We explored potential sources of bias in ΔPTPaw-based estimates. A longer insufflation time (Ti) was significantly associated with increased disagreement between ΔPTPaw and PTPdi. One possible explanation is that extended Ti allow for more sustained airway pressurization by the ventilator, reducing the relative drop in airway pressure during stimulated breaths and thereby underestimating diaphragmatic effort. Additionally, longer Ti may lead to activation of expiratory muscles toward the end of inspiration, which could reduce transdiaphragmatic pressure and thus PTPdi. Interestingly, this effect was not observed for PTPmus, possibly due to the more direct correspondence with pleural pressure dynamics. Additionally, higher lung resistance (Rlung) was significantly associated with greater bias between ΔPTPaw and PTPmus. This likely reflects the attenuation of pressure transmission across resistive airways, which may decouple airway pressure from actual pleural pressure changes. These findings suggest that ΔPTPaw may be less accurate under conditions of high Rlung or prolonged insufflation time, reinforcing the need for context-specific interpretation when using this surrogate in clinical settings. In our analysis, PEEP did not significantly influence the relationship between ΔPTPaw and diaphragmatic effort. Within the PEEP range used in this study, neurostimulation-induced diaphragmatic contraction appeared largely independent of PEEP level.

Although ΔPTPaw currently requires offline signal analysis, some commercially available ventilator monitoring systems and DNAV devices already incorporate automated detection of airway pressure-time product changes, which could facilitate real-time bedside application of this method in the future.

### Limitations

This study has several limitations. First, the sample size was relatively small, and most participants were post-surgical patients, which may limit the generalizability of our findings to broader ICU populations with more severe or heterogeneous forms of respiratory failure.

Second, although a substantial number of cycles were analyzed—with 63% classified as adequate for analysis—over one-third were excluded due to signal artifacts or acquisition issues. To ensure internal validity, only artifact-free cycles with optimal signal quality were included, which may have reduced statistical power but strengthened the reliability of the observed correlations, especially the strong relationship between ΔPTPaw and PTPmus. Additionally, the validation against transdiaphragmatic pressure (Pdi) was limited by the availability of interpretable Pdi signals in only nine patients.

Third, the thresholds used to define low and excessive diaphragmatic effort were based on physiologic studies in healthy individuals and may not accurately reflect safe or optimal loading targets in critically ill patients. The long-term impact of different stimulation intensities—particularly in vulnerable muscle conditions—remains to be clarified.

Forth, the technique described is only valid during passive ventilation in assist-controlled volume-controlled mode. In pressure-targeted modes, elicited diaphragm contractions would increase tidal volume, so the magnitude of diaphragmatic pressure generation cannot be directly estimated. For this reason, volume-targeted ventilation may be preferable during DNAV to ensure constant tidal volume and permit monitoring of diaphragmatic loading.

## Conclusion

Diaphragm neurostimulation exhibits a dose-response relationship with diaphragm contractility. ΔPTPaw is a reliable, non-invasive surrogate for monitoring diaphragm loading during DNAV under assist-controlled volume-controlled mode and may guide neurostimulation titration in mechanically ventilated patients.

## Supplementary Information

Below is the link to the electronic supplementary material.

Supplementary Material 1.

## Data Availability

The datasets generated and analyzed during the current study are not publicly available due to privacy and institutional restrictions but are available from the corresponding author on reasonable request.

## Abbreviations

DNAV: Diaphragm neurostimulation-assisted ventilation
ΔPTPaw: Reduction in airway pressure-time product
PTPdi: Transdiaphragmatic pressure-time product
PTPmus: Respiratory muscles pressure-time product
PEEP: Positive end-expiratory pressure
ROC: Receiver operating characteristic
AUROC: Area under receiver operating characteristic curve
MV: Mechanical ventilation
DNS: Diaphragm neurostimulation
Paw: Airway pressure
Pes: Esophageal pressure
Pga: Gastric pressure
Pdi: Transdiaphragmatic pressure
Pmus: Respiratory muscles pressure
PTP: Pressure-time product
RR: Respiratory rate
PADMER: Program for Advanced Analysis of Respiratory Mechanics
IP: Intellectual property
Rlung: Lung resistance
EL: Lung elastance
Eab: Abdominal elastance
Ti: Insufflation time
Pocc: Occlusion pressure
