# Supplementary material for: Monitoring diaphragmatic effort during diaphragm neurostimulation-assisted ventilation
Source: Crit Care. 2025 Dec 23;30:20. doi: 10.1186/s13054-025-05810-z (PMC12790116; doi:10.1186/s13054-025-05810-z)
Supplement: Supplementary file 1 — Supplementary Material 1. [file 13054_2025_5810_MOESM1_ESM.docx]

**Online Data Supplement**

**Monitoring Diaphragmatic Effort During Neurostimulation-Assisted Ventilation**

**Supplemental Description of the Methods**

*Study design and setting*

As part of the STIMULUS trial, we conducted a nested randomized cross-over trial testing the effect of progressively increasing doses of diaphragm neurostimulation at two levels of positive end-expiratory pressure (PEEP). This study was conducted over a 2-hour period shortly after enrolled patients met criteria to initiate stimulation and device mapping was successfully completed. Details on the study population and methods have previously been reported in detail.^1^ Of the 19 enrolled patients, three did not undergo the titration protocol because of ongoing neuromuscular blockade, inability to stimulate the diaphragm after initial successful mapping, or insufficient diaphragm contractile response to stimulation.

*Study Procedures*

During the nested titration study, patients were randomized to lower or higher positive end expiratory pressure (PEEP) in a cross-over design. Lower PEEP was defined as the lowest level of PEEP tolerated in terms of maintaining oxygenation, titrated to a minimum of 5 or 10 cm H_2_O in the surgical and AHRF groups respectively. Higher PEEP was defined as a PEEP sufficient to achieve an end-expiratory transpulmonary pressure ≥0 cm H_2_O and at least 5 cm H_2_O higher than the selected lower PEEP level. Diaphragm neurostimulation was progressively increased in four steps from absent stimulation at baseline (Pocc 0 cm H_2_O), followed by stimulation levels targeting Pocc –5, –10, and –15 cm H_2_O. Each step was maintained for 10 minutes before measurements were obtained. This sequence of steps was repeated at higher and lower levels of PEEP, applied in a random order. Stimulation intensity was modulated by adjusting electrical current (up to 27 mA), frequency (15–40 Hz), or stimulation duration (0.1–1.2 seconds), to achieve targeted Pocc levels.

*Study measurements and signal analysis*

For each stimulation phase (at high and low PEEP) in each patient, the following analysis procedure was applied:

1. **Selection of Passive Breaths:** A representative unstimulated breath was manually selected to characterize fully passive pulmonary mechanics.
2. **Selection of Stimulated Breaths:** High-quality stimulated breaths were manually screened by two independent reviewers (ACF and FPG). Ten breaths were selected per stimulation level (-5, -10, and -15 cm H₂O target Pocc) per phase.
3. **Signal Pre-processing:** Raw respiratory signals were pre-processed using a custom, computer-based algorithm developed ad hoc for this study. For each breath, the algorithm automatically identified the start of inspiration, the start of expiration, and the end of expiration based on the flow signal. These landmarks were used to segment individual breaths and enable temporal alignment. Each stimulated breath was then time-aligned with the preceding passive breath, ensuring consistent phase matching for subsequent breath-by-breath comparisons.
4. **Differential Calculations:** After synchronization, inspiratory and expiratory phases were used to compute pressure differences and time-based integrals. For the expiratory phase, measurements were referenced 0.5 seconds before the end of the respiratory cycle to ensure analysis during a stable expiratory period.
5. **Quality Control and Exclusions:** Graphical visualizations of each processed signal were generated for quality verification. Breaths with inadequate signal quality for any required measurement, despite prior selection, were excluded from the final analysis.

**REFERENCES**

1. Morris IS, Bassi T, Bellissimo CA, et al. Continuous On-Demand Diaphragm Neurostimulation to Prevent Diaphragm Inactivity During Mechanical Ventilation: A Phase 1 Clinical Trial (STIMULUS). *Am J Respir Crit Care Med*. Published online March 5, 2025. doi:10.1164/rccm.202407-1483OC

**TABLES**

**e-Table 1.** Predictors of Bias Between ΔPTPaw and Diaphragmatic Effort Estimates.

| **Outcome** | **Predictor** | **Estimate (β)** | **95% CI** | **p-value** |
| --- | --- | --- | --- | --- |
| ΔPTPaw - PTPdi (per breath) | Lung resistance | -0.20 | -0.41 to 0.03 | 0.095 |
|  | Lung elastance | 0.92 | -1.49 to 3.33 | 0.441 |
|  | Insufflation time | 6.12 | 0.92 to 11.32 | **0.022** |
|  | Stimulation level | -0.23 | –0.59 to 0.13 | 0.202 |
|  | Abdominal elastance | -0.00 | –0.01 to 0.01 | 0.569 |
| ΔPTPaw - PTPmus (per breath) | Lung resistance | -0.28 | –0.43 to –0.13 | **0.001** |
|  | Lung elastance | 0.49 | –1.16 to 2.13 | 0.551 |
|  | Insufflation time | 1.63 | –2.02 to 5.28 | 0.370 |
|  | Stimulation level | -0.01 | –0.23 to 0.21 | 0.926 |
|  | Abdominal elastance | 0.01 | –0.00 to 0.03 | 0.137 |

Estimates derived from univariable linear mixed-effects models with patient as random intercept. Significant p-values shown in bold.

*ΔPTPaw = difference in airway pressure-time product between stimulated and non-stimulated breaths; PTPdi =, transdiaphragmatic pressure-time product; PTPmus = respiratory muscle pressure-time product.*
